# The child who lived: Down syndrome among Neanderthals?

**DOI:** 10.1126/sciadv.adn9310

**Published:** 2024-06-26

**Authors:** Mercedes Conde-Valverde, Amara Quirós-Sánchez, Julia Diez-Valero, Nieves Mata-Castro, Alfredo García-Fernández, Rolf Quam, José Miguel Carretero, Rebeca García-González, Laura Rodríguez, Ángeles Sánchez-Andrés, Juan Luis Arsuaga, Ignacio Martínez, Valentín Villaverde

**Affiliations:** ^1^ Universidad de Alcalá, Departamento de Ciencias de la Vida, Cátedra de Otoacústica Evolutiva y Paleoantropología (HM Hospitales-Universidad de Alcalá), Alcalá de Henares, Spain.; ^2^ Department of Anthropology, Binghamton University (SUNY), Binghamton, NY, USA.; ^3^ Hospital Universitario HM Puerta del Sur, Móstoles, Spain.; ^4^ Hospital Universitario HM Montepríncipe, Boadilla del Monte, Spain.; ^5^ Hospital Universitario 12 de Octubre, Madrid, Spain.; ^6^ Centro de Investigación UCM-ISCIII sobre la Evolución y Comportamiento Humanos, Madrid, Spain.; ^7^ Division of Anthropology, American Museum of Natural History, New York, NY, USA.; ^8^ Laboratorio de Evolución Humana, Universidad de Burgos, Burgos, Spain.; ^9^ Unidad Asociada de I+D+i al CSIC Vidrio y Materiales del Patrimonio Cultural (VIMPAC), Universidad de Burgos, Burgos, Spain.; ^10^ Área de Antropología Física. Departamento de Biodiversidad y Gestión Ambiental, Universidad de León, Facultad de Ciencias Biológicas y Ambientales, Campus De Vegazana, León, Spain.; ^11^ Departamento de Geodinámica, Estratigrafía y Paleontología, Facultad de Ciencias Geológicas, Universidad Complutense de Madrid, Madrid, Spain.; ^12^ Universitat de València, Departament de Prehistòria, Arqueologia i Història Antiga (PREMEDOC), Av. Blasco Ibañez 28, 46010 València, Spain.

## Abstract

Caregiving for disabled individuals among Neanderthals has been known for a long time, and there is a debate about the implications of this behavior. Some authors believe that caregiving took place between individuals able to reciprocate the favor, while others argue that caregiving was produced by a feeling of compassion related to other highly adaptive prosocial behaviors. The study of children with severe pathologies is particularly interesting, as children have a very limited possibility to reciprocate the assistance. We present the case of a Neanderthal child who suffered from a congenital pathology of the inner ear, probably debilitating, and associated with Down syndrome. This child would have required care for at least 6 years, likely necessitating other group members to assist the mother in childcare

## Teaser

A Neanderthal child with a debilitating pathology required social care to survive for at least 6 years.

## INTRODUCTION

The existence of caregiving for sick or injured individuals among Neanderthals has been known for a long time, although interest in understanding the implications of this behavior has increased in recent years (*1*–*4*). For some authors, the caregiving among Neanderthals would be related to a broader and more complex social context, of great adaptive value (*3*–*8*). Here, we present the case of a Neanderthal individual who survived to at least 6 years of age suffering from a severe inner ear pathology, most likely associated with the presence of Down syndrome. The symptoms produced by this pathology would have included, at a minimum, severe hearing loss and markedly reduced sense of balance and equilibrium. Thus, the care necessary for their survival over a period of several years likely exceeded the capabilities of the mother and would have required the help of other members of the social group. This is a known case in Neanderthals of social care for a child with a severe pathology.

An interesting debate in the field of bioarchaeology is about the existence of care in prehistory based on the study of lesions in fossil specimens. On one hand, some authors (*9*–*11*) argue that it is not possible to rigorously infer the existence of care from mere paleopathological evidence and that the inferences made are based on unwarranted assumptions. In recent years, however, the idea that paleopathological evidence is an objective source of information on the existence of care in prehistory has been gaining ground, and a method for making systematic inferences about the nature of care from paleopathological evidence has already been proposed (*3*, *6*).

Another particularly interesting aspect in the field of bioarchaeology of care applied to fossil hominins is to determine why people devoted part of their time and effort to care for a temporarily or permanently disabled member of their group. On the one hand, there are authors who believe that caregiving took place in a context of reciprocal selfishness between individuals able to reciprocate the favor, while other authors argue that assistance to sick or injured individuals among Neanderthals went beyond reciprocal selfishness and was produced by a genuine feeling of compassion (*1*–*4*, *6*, *7*). In turn, this compassionate feeling would be part of a broader collaborative behavior of great adaptive value that would include other prosocial behaviors (*1*, *3*–*8*). In this context, the study of infants with pathologies that would have required the assistance of other members of the group to survive is particularly interesting, as children have a very limited possibility to reciprocate the assistance received. On the other hand, when the intensity or duration of the care necessary for the child’s survival exceeds the mother’s possibilities, she requires additional extramaternal help from other individuals in the social group. Thus, the study of individual children offers the possibility of testing whether the caregiving is directly related to such a complex social strategy as collaborative parenting (*4*, *12*). Unfortunately, to date, no case of a pathological immature Neanderthal individual has been known for which these hypotheses could be tested. In this context, fossil CN-46700 from the Cova Negra site is of particular interest because it is a Neanderthal child with signs of having suffered a severe congenital pathology, and its study may help us to better understand the extent of social care among Neanderthals.

### Cova Negra site

**Fig. 1.**
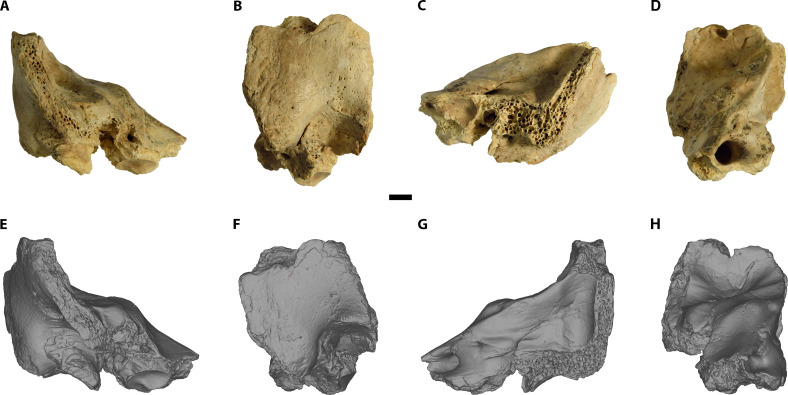
Original fossil and 3D model of CN-46700. (**A** to **D**) original fossil. (**E** to **H**) 3D model. [(A) and (E)] anterior view. [(B) and (F)] Lateral view. [(C) and (G)] Posterior view. [(D) and (H)] Medial view. Scale bar, 5 mm.

Cova Negra is a cave site in the locality of Xàtiva (Valencia, Spain) and has been excavated at different periods between 1929 and 2017 (*13*). A set of human fossil remains are known from this site from levels dated by electron spin resonance (ESR)/U-series between 273 and 146 thousand years ago (ka) (*14*) and have been assigned to the species *Homo neanderthalensis* (*15*). Recently, when sorting through the faunal remains from the 1989 campaign, three new fragments of human fossils were found, deriving from an undated disturbed level at the site (fig. S1) that contained archaeological materials belonging mostly from the Middle Paleolithic (77.2%), although it also includes materials from the Upper Paleolithic (22.7%). Among these new remains is a fragment of the right petromastoid region of an immature temporal bone (CN-46700) (Fig. 1). Given its provenience from a disturbed level possibly containing either *H. neanderthalensis* or *Homo sapiens* fossils, the first step of our study focused on a taxonomic identification.

## RESULTS

### Taxonomic diagnosis

**Fig. 2.**
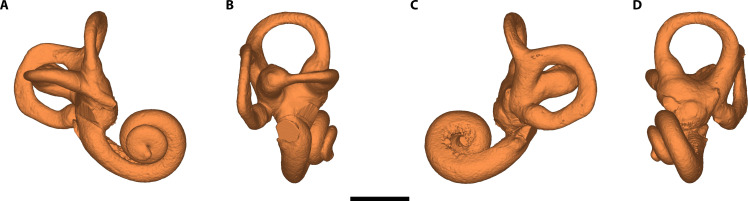
3D model of the inner ear of CN-46700. (**A**) Anterior view. (**B**) Lateral view. (**C**) Posterior view. (**D**) Medial view. Scale bar, 5 mm.

Micro–computed tomography (μCT) scans of the original fossil were used to reconstruct a three-dimensional (3D) model for measurement and analysis (Fig. 2).

For our analysis, we have used six taxonomically relevant inner ear characteristics to differentiate Neanderthals from *H. sapiens*. Four of them correspond to the semicircular canals (*16*): the relative placement of the posterior semicircular canal (SLI) and the radii of the anterior semicircular canal (ASC-R), posterior semicircular canal (PSC-R), and lateral semicircular canal (LSC-R). The other two variables used correspond to the cochlea (*17*): the total number of cochlear turns (NT), and the proportional length of the third cochlear turn (%L3)*.*

Because CN-46700 has an abnormally reduced cochlear volume (see below), it would be reasonable to think that this reduction could affect the NT and %L3 variables and distort the results of the taxonomic analysis. To establish whether variations in cochlear volume affect NT and %L3 variables, we calculated the correlation coefficients between these variables in a sample of modern humans (data S1). Our results demonstrate that cochlear volume is neither correlated with the variable NT (*r* = 0.1341, *P* = 0.480) nor with the variable %L3 (*r* = 0.0852, *P* = 0.654), indicating that variations in cochlear volume do not affect NT and %L3 values.

The value in CN-46700 for SLI is similar to the mean of our Neanderthal sample but is about 2 *z*-scores away from the sample means of three modern human and one *H. sapiens* fossil used for comparison (Table 1). On the other hand, the NT and %L3 values of the Cova Negra sample fall within the range of variation of the Neanderthals but are outside the range and more than 2 *z*-scores away from the mean of our modern *H. sapiens* sample (Table 1 and fig. S2).

**Table 1.** Taxonomically diagnostic variables of the inner ear in CN-46700, Neanderthals, and *H. sapiens*. SLI, sagittal labyrinthine index; NT, number of turns of the cochlea; %L3, proportional length of the third turn of the cochlea. For individual values, see tables S1 and S2.

	CN-46700		*H. neanderthalensis*	*H. sapiens*	*H. sapiens fossils*
SLI	63.10	Mean ± SD (*z*-scores)	63.4 ± 5.4 (−0.05)	51 ± 7* (1.73)	49.6 ± 7.1 (1.89)
				50.6 ± 5.4† (2,31)	
				47.8 ± 6.4‡ (2.39)	
		Min – max (*n*)	53.0–76.0 (28)	34–39 (54)*	33.0–62.5 (25)
				38.9–61.1 (26)†;	
				36.8–62.4 (110)‡	
NT	2.22	Mean ± SD (*z*-scores)	2.37 ± 0.14§ (−1.07)	2.58 ± 0.14 (−2.65)	
		Min – max (N)	2.18–2.59 (7)§	2.33–2.87 (30)	
%L3	8.58	Mean ± SD (*z*-scores)	9.4 ± 3.5§ (−0.23)	14.40 ± 2.35 (−2.47)	
		Min – max (*n*)	5.2–15.1 (7)§	9.7–18.2 (30)	

*Data from (*16*).

†Data from (*94*).

‡Data from (*95*)

§Data from (*17*).

**Fig. 3.**
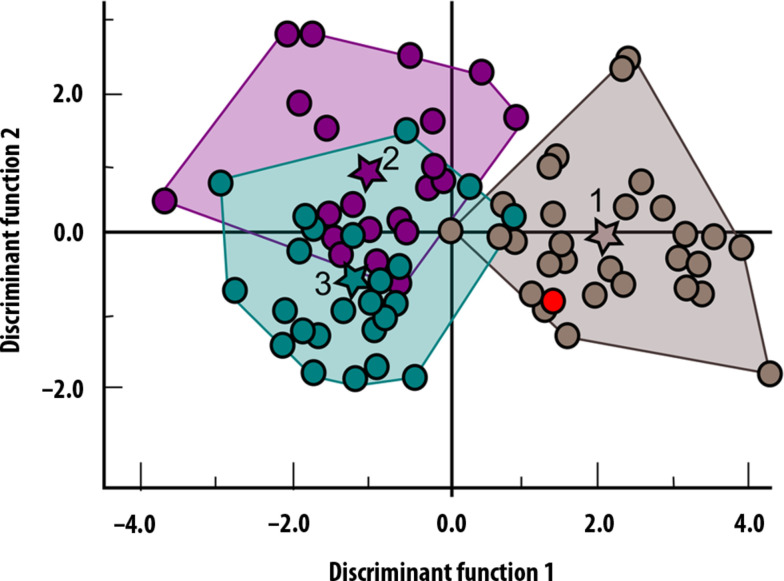
Canonic discriminate function graph. Brown, *H. neanderthalensis*; green, recent *H. sapiens*, and purple, fossil *H. sapiens*; dots, individuals in the analysis. Star centroid group (1: *H. neanderthalensis*; 2: Fossil *H. sapiens*; 3: Recent *H. sapiens*). Red dot represents CN-46700 specimen.

To complete our taxonomic analysis of CN-46700, we performed a discriminant function analysis with the four semicircular canal variables (SLI, ASC-R, PSC-R, and LSC-R) on a sample of 29 Neanderthals, 24 fossil *H. sapiens*, and 26 extant *H. sapiens* (data S2). The results of our analysis allow us to distinguish between *H. sapiens* and *H. neanderthalensis* in almost all cases (Fig. 3 and tables S3 to S5). The Cova Negra specimen is assigned to Neanderthals with a probability of 94%. In our opinion, these results sufficiently justify the assignment of CN-46700 to *H. neanderthalensis*.

### Age at death

**Fig. 4.**
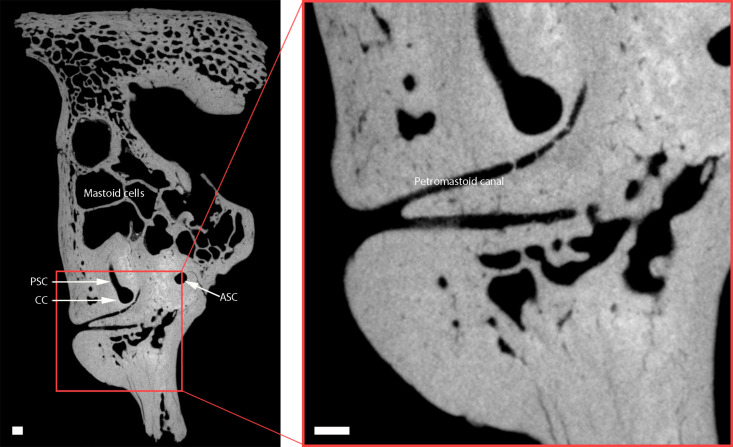
Petromastoid canal of CN-46700 in an axial view. CC, common crus; PSC, posterior semicircular canal; ASC, anterior semicircular canal. Scale bar, 1 mm.

In CN-46700, the subarcuate fossa is obliterated, and the presence of a petromastoid canal is observed (Fig. 4). This situation occurs in modern human populations between 2 to 5 years of age (*18*, *19*). The dimensions of the petromastoid canal also vary with age, and the value in CN-46700 (0.29 mm wide) corresponds to an age of death of older than 6 years according to recent human standards (*20*). The recent study of the skeleton of a Neanderthal individual aged 6 to 7 years at death indicates an overall growth rate in Neanderthals similar to that of modern human children (*21*). Although the study of the El Sidrón skeleton does not include the petrous bone, it seems reasonable to extrapolate that the growth rate of the petrous bone could also be similar to that of modern humans on the basis of the results from other bones. In that case, an age at death greater than 6 years to the individual represented by CN-46700 seems plausible.

### Pathological evidence

An anomalous dilatation of the lateral semicircular canal (LSC), which is more marked in the nonampullary limb, is observed in the μCT images of CN-46700 (Fig. 2). This dilatation affects the entire arch of the canal, as demonstrated by the reduced dimensions of the bony island and the increased cross-sectional area along the entire length of the LSC, whose values are outside the range of variation of the Neanderthal sample (Table 2, fig. S3, and data S3) and can be considered as a case of LSC dysplasia (*22*). The anterior semicircular canal (ASC) of CN-46700 also shows a reduced bony island compared to the other Neanderthal specimens, although in this case, there is no increase in the cross section of the canal lumen (Table 2). This indicates that the ASC of CN-46700 has a hypoplastic bony island (*23*). Last, the posterior semicircular canal (PSC) is of similar dimensions to those of the rest of the Neanderthal sample (Table 2) and can therefore be considered normal.

**Table 2.** Pathologically diagnostic variables of the inner ear in CN-46700 and Neanderthals. LSC, lateral semicircular canal; ASC, anterior semicircular canal; PSC, posterior semicircular canal; VA, vestibular aqueduct; V, vestibule; C, cochlea; CNC, cochlear nerve canal. See data S2 for individual value of the Neanderthal sample.

	CN-46700	*Z*-scores	*H. neanderthalensis* (*n* = 6)
			mean ± SD (min – max)
LSC-Bony island area (mm^2^)	6.94	−4.84	12.55 ± 1.16 (11.06–14.08)
LSC-Maximum diameter of the bony island (mm)	3.54	−2.58	4.34 ± 0.31 (4.02–4.80)
LSC-Canal lumen area at the ampullar end (mm^2^)	3.22	0.52	2.96 ± 0.50 (2.03–3.33)
LSC-Canal lumen area at the nonampullar end (mm^2^)	4.48	4.45	2.12 ± 0.53 (1.37–2.85)
LSC-Canal lumen area at the apex (mm^2^)	1.21	1.67	0.81 ± 0.24 (0.42–1.17)
ASC-Bony island area (mm^2^)	15.30	−2.96	20.80 ± 1.86 (18.06–23.08)
ASC-Maximum diameter of the bony island (mm)	5.05	−3.87	5.63 ± 0.15 (5.36–5.82)
ASC-Canal lumen area at the ampullar end (mm^2^)	2.98	0.31	2.79 ± 0.62 (1.64–3.48)
ASC-Canal lumen area at the nonampullar end (mm^2^)	1.62	−0.95	1.83 ± 0.22 (1.61–2.18)
PSC-Bony island area (mm^2^)	12.60	−0.73	15.04 ± 3.33 (10.35–19.02)
PSC-Maximum diameter of the bony island (mm)	4.54	−0.31	4.64 ± 0.32 (4.03–4.94)
VA-Width at midpoint between common crus and operculum (mm)	3.38	29.3	0.45 ± 0.10 (0.30–0.61)
VA-Width at operculum (mm)	6.65	2.05	3.49 ± 1.54 (2.07–5.85)
VA-Volume (mm^3^)	19.58	4.21	4.28 ± 3.63 (1.12–10.05)
V-Maximum width (mm)	3.03	−2.00	3.41 ± 0.19 (3.17–3.67)
V-Maximum height (mm)	6.01	−2.08	6.53 ± 0.25 (6.20–6.77)
V-Area (mm^2^)	14.58	−2.14	17.87 ± 1.54 (16.30–20.00)
C-Volume (mm^3^)	64.47	−1.79	79.44 ± 8.38 (65.14–102.00)*
CNC-Width (mm)	2.34	−0.14	2.37 ± 0.22 (2.07–2.68) (*n* = 5)

*n = 15. See data S2 for individual value of the Neanderthal sample.

**Fig. 5.**
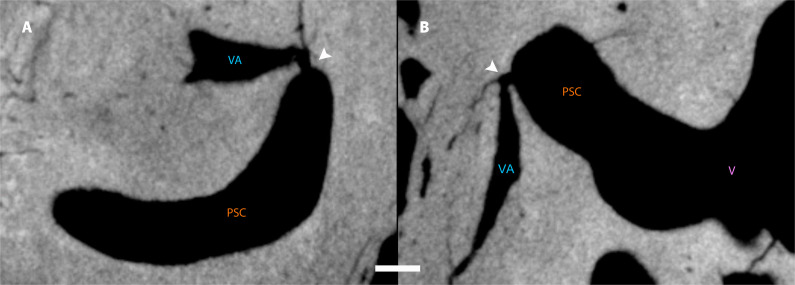
Fistula between the posterior semicircular canal and the vestibular aqueduct in CN-46700. (**A**) Sagittal view. (**B**) Axial view. The white arrows in (A) and (B) indicate the position of the fistula. Scale bar, 1 mm.

In modern humans, the presence of LSC dysplasia is often accompanied by other inner ear malformations, the most common being the presence of an enlarged vestibular aqueduct (EVA) (*24*–*26*). The dimensions of the vestibular aqueduct (VA) in CN-46700 are much larger than those of the specimens from our Neanderthal sample (Table 2 and fig. S4), which clearly indicates the existence of EVA in CN-46700. In addition, a small communication, or fistula (Fig. 5), is visible between the PSC and the VA of CN-46700 (fig. S5). Although the most common labyrinth fistulas are those connecting the labyrinth to the middle ear, fistulas connecting the semicircular canals to the VA have also been described (*27*). The most common cause of fistulae in the semicircular canals is acute otitis media complicated by labyrinthitis (*28*, *29*).

LSC dysplasia also often occurs in association with other alterations in the cochlea and in the vestibule. In the case of the cochlea, there is a reduction in the overall size of the cochlea (*22*, *23*), and Mondini malformation (absence of the modiolus) may also occur (*30*). The cochlea of CN-46700 does not show Mondini malformation, but the cochlear volume is small, outside the range of the Neanderthal sample, and at −1.79 *z*-scores from its mean (Table 2). In turn, vestibule alterations associated with LSC dysplasia generally consist of an increase in its dimensions (*22*, *30*), although in some syndromes (see below) the opposite may occur, and the vestibule becomes smaller (*31*). The dimensions of the vestibule of CN-46700 are below the range of the Neanderthal sample, clearly indicating that its vestibule is reduced (Table 2). Last, LSC dysplasia may also be associated with cochlear canal stenosis and/or alterations in the facial nerve pathway (*32*, *33*). The dimensions of the cochlear duct of CN-46700 are similar to those of the other Neanderthal specimens in the sample (Table 2), which rules out stenosis of the bony canal of the cochlear nerve, and the pathway of the facial nerve as it passes the cochlea is also normal (fig. S5).

### Symptomatology associated with pathologies

LSC dysplasia is the most common congenital inner ear disorder (*34*). Although it can be asymptomatic (*35*), it most commonly is associated with sensorineural or mixed (sensorineural and conductive) hearing loss (*36*, *37*) in addition to vestibular symptoms such as loss of balance and severe vertigo (*34*, *36*–*38*). A dilated VA is also congenital and can occur in isolation or in association with other disorders of the inner ear or adjacent structures (*26*, *38*–*41*). This malformation is always associated with severe sensorineural hearing loss (*24*, *38*, *40*, *42*) and with the presence of vestibular symptoms (*42*–*44*). Last, abnormal communication between the PSC and the VA also causes vestibular symptoms such as third window syndrome and/or Tullio’s phenomenon (*45*, *46*), with vertigo produced by loud sounds or pressure changes. In summary, the set of pathologies present in CN-46700 produced a lifelong symptomatology that would include severe hearing loss, and frequent crises of acute disabling vertigo and imbalance.

## DISCUSSION

The occurrence in the same individual of such a wide range of malformations as those presented by CN-46700 indicates the presence of a syndrome that is compatible with all of them. Syndromes that include LSC dysplasia and/or EVA include CHARGE syndrome (*47*), Pendred syndrome (*48*, *49*), branchio-otorenal syndrome (*32*), Waanderburg syndrome (*50*), Allagile syndrome (*51*), and Down syndrome (*31*)*.*

Aplasia of the semicircular canals is generally considered a major criterion for diagnosis of CHARGE syndrome (*52*, *53*), so the presence of all three semicircular canals in CN-46700 allows CHARGE syndrome to be confidently discarded. Waanderburg syndrome is also characterized by aplasia of one of the semicircular canals (*50*, *54*), as well as by the presence of an enlarged vestibule (*54*), criteria that are not met in the case of CN-46700, which allows us to rule out this syndrome as well. Alagille syndrome includes partial or total agenesis of the PSC (*55*, *56*), and, because CN-46700 has a normal PSC, we can also discard this syndrome. The presence of a normal modiolus in CN-46700 also allows us to discard Pendred syndrome (*37*, *48*). Last, the presence of branchio-otorenal syndrome can also be ruled out because both the dimensions of the cochlear nerve canal and the facial nerve pathway are normal (*32*, *33*).

The only syndrome that is compatible with the entire set of malformations present in CN-46700 is Down syndrome, where LSC dysplasia is common (*31*, *57*), both in isolation and accompanied by EVA (*26*). Other malformations present in CN-46700 are also common in Down syndrome, such as a hypoplastic ASC (*58*) and a small cochlea (*31*, *57*, *58*). Moreover, although hypoplasia of the vestibule has also been described in CHARGE syndrome (*52*), the association between LSC dysplasia and the presence of a reduced vestibule has only been reported in individuals with Down syndrome (*31*, *57*–*59*).

Consequently, all available evidence suggests that the CN-46700 individual probably had Down syndrome, which is the most common human genetic disorder (*60*), and it is also present in great apes (*61*, *62*). Down syndrome is associated with a wide variety of impairments (*60*) in addition to those produced by the inner ear malformations. These alterations affect growth and physical and cognitive development (*63*–*65*), which has a strong impact on all stages of child development up to adulthood, leading to delays in ambulation (*66*) and speech acquisition (*67*), relative to the population in their age range. Instrumental learning and the development of communication and social skills are also affected (*63*, *65*). One of the impairments that is present in more than 80% of infants with Down syndrome is generalized hypotonia (*68*, *69*) which has an important impact on breastfeeding due to poor sucking strength (*70*–*72*) that prevents an adequate oral seal during breastfeeding. In addition, children with Down syndrome have delayed psychomotor development (*73*) due to a combination of factors including, in addition to generalized hypotonia, ligament hyperlaxity, poor postural control, and poor balance (*74*). This results in a substantial delay in gross motor development that manifests in delayed ambulation and lack of motor coordination and balance, which increases the risk of falls and hinders exploratory learning (*75*, *76*).

Because children with Down syndrome have delayed tooth eruption and general skeletal growth (*77*), they may also have delayed age of subarcuate fossa closure. In that case, the individual represented by CN-46700 would have a minimum age at death of more than 6 years.

A case is known of a chimpanzee with Down syndrome that survived to 23 months of age thanks to the care received by the mother, who was assisted by the eldest daughter (*62*). When the daughter stopped helping the mother to care for her own offspring, the mother was unable to provide the necessary care and the offspring died. On the other hand, the oldest cases of Down syndrome in our species have been documented by Rohrlach *et al.* (*78*), who identified five cases of Down syndrome in individuals from prehistoric populations (from 400 BCE to 3629 BCE). None of these individuals survived beyond 16 months of extrauterine life. These data show the short life expectancy of children with Down syndrome in nature and in prehistoric times. It is therefore notable that the individual represented by fossil CN-46700 lived to at least 6 years of age, which far exceeds the usual life expectancy of children with Down syndrome in prehistoric population.

It is reasonable to think that the long survival of individual CN-46700 could only have occurred because it received continuous care and attention during that time. The life expectancy of children with Down syndrome can be increased if they receive the necessary care and attention. Thus, in 1929, the life expectancy of children with Down syndrome, which was only 9 years (*61*), increased to 12 years in the 1940s (*79*) and now exceeds 60 years in developed countries (*79*). This marked increase in the life expectancy of people with Down syndrome is undoubtedly due, in large part, to advances in medicine but also to social changes that have led advanced societies to increase social protection for children with Down syndrome and their families.

Given the chronic nature of their impairments, the individual represented by CN-46700 would have required continuous and important care, beyond the normal altricial care, throughout their life. Because of the demanding lifestyle of Neanderthals, including high levels of mobility (*4*, *6*, *7*), it is difficult to think that the mother of the individual would have been able to provide such care alone and also carry out normal daily activities over a prolonged period of time. It is likely, therefore, that the mother required the continuous help of other members of the social group, either for assistance in performing other daily tasks (or to relieve her from performing them) or to directly assist in providing the necessary care for the child, or both.

Other cases are known in the fossil record of individuals with inner ear pathologies resulting in hearing loss and impaired balance. The most relevant cases are Singa (*80*) and Dar-Es- Soltane II H5 (*81*). However, unlike CN-46700, both fossils correspond to adult individuals who suffered from labyrinthitis ossificans, a pathology that is not congenital.

The case of the CN-46700 individual it is particularly interesting because social care was destined to an immature individual who had no possibility to reciprocate the assistance received. There are also two known cases of immature individuals with pathologies that may have caused neurological disorders. One is the case of Qafzeh 11, a specimen of *H. sapiens*, dated to around 90 to 100 ka, which suffered a severe cranial trauma that may have caused neurological disorders (*82*). The other case is that of a pre-Neanderthal preadolescent (Cranium 14) from the Sima de los Huesos site, who suffered from congenital craniosynostosis that deformed the skull and face (*83*). However, the neurological extent of this pathological condition has not yet been established with certainty in Cranium 14 (*1*, *7*). Therefore, in the case of Cranium 14 from Sima de los Huesos, it is not possible to reliably determine the extent of care these individuals needed to survive.

Last, the evidence provided by CN-46700 is fully compatible with the idea previously advocated by other authors that caregiving and collaborative parenting occurred together in Neanderthals and that both prosocial behaviors were part of a broader social adaptation of high selective value that must have been very similar to that of our species (*1*–*4*, *6*, *7*). Moreover, the presence of this complex social adaptation in both Neanderthals and our own species suggests a very ancient origin within the genus *Homo* (*5*, *8*).

## MATERIALS AND METHODS

### Taxonomic analysis

For the discriminant function analysis (Supplementary Text), we used data on SLI, ASC-R, LSC-R, and PSC-R variables from the literature for 29 Neanderthals, 23 fossil *H.* sapiens, and 26 modern humans (data S2). We also used the original data for the cochlear variables NT and %L3 from 10 Spanish medieval individuals and 20 British medieval specimens (data S1). The discriminant function analysis has been performed with IBM SPSS v.29.

### Pathological analysis

To establish whether the values of CN-46700 in the diagnostic variables fall within the variability of Neanderthals or can be considered pathological, we have compared it with a sample of Neanderthals from sites in France (La Chapelle-aux-Saint, La Quina, and La Ferrassie) and Israel (Amud and Kebara):

La Chapelle-aux-Saint 1 is a skull of an adult individual, and the right temporal bone was used in this study. The dates for the site layer from which the fossil originated was conducted, using two different methods: ESR (47 ± 3 ka) and thermoluminescence (56 ± 4 ka) (*84*).

La Quina H5 is a calvarium of an adult individual that preserves the left temporal bone. The occupation of the site of La Quina is attested to have occurred from 63 to 55 ka until 40 ka (*85*).

La Ferrassie 1 was assigned to an adult male individual, while La Ferrassie 2 was assigned to an adult female individual. The layer where both crania were found has been dated to 39.7 ± 2.3 ka (*86*). Amud 1 is a skull of an adult individual, including the complete left temporal bone, which has been dated to 53 ± 8 thousand years (*87*).

Kebara 1 has been attributed to a 7- to 9-month-old infant. The site of Kebara Cave has been dated to 60 ± 6 ka (*88*).

### CT scanning

Micro-CT scanning of the CN-46700 was carried out at the Museo Nacional de Ciencias Naturales in Madrid, Spain using a CT-SCAN- XT H-160 (NIKON) micro-CT scanner. A total of 1535 slices were obtained as a 1145 × 1075 matrix and saved in Digital Imaging and Communication In Medicine format. Scanning parameters were the following: isometric voxel size of 0.034 mm, field of view = 39.01 mm, voltage = 160 kV, and current = 226 μA. Virtual reconstruction of the bony labyrinth was made using the Mimics software program.

### Measurement protocol

All the measurements used in this study were taken on the 3D model of CN-47600. Some of the measurement protocols have been taken from previous studies, while on other occasions, it has been necessary to develop specific protocols for the present study. A detailed description and diagrams of the protocols followed to quantify the pathologically diagnostic variables are given in data S4.

The Sagittal Labyrinthine Index was measured using the protocol described by Spoor *et al.* (*16*). The number of turns of the cochlea and the proportional length of the third turn were measured using the protocol described by Conde-Valverde *et al.* (*17*). To estimate the age at death of individual CN-46700, we applied the method used by Skrzat *et al.* (*89*) to measure the maximum diameter in the petromastoid canal section.

For the measurements of the area of the bony island of the three canals and the maximum diameter of the bony island of the ASC and of the PSC, we developed protocols for this study. The area of the bony island was measured in the midline plane of each semicircular canal, and the maximum diameter was taken from the nonampullary part in the same plane in which the area was measured in each canal. For the maximum diameter of the LSC bony island, the procedure of Purcell *et al.* (*22*) was followed.

The cross-sectional area of the lumen of the lateral and ASCs in the ampullary zone and in the nonampullary zone was also measured using a protocol designed for this study that consists of placing a transverse plane to the corresponding canal and measuring the area of the cut section. The cross-sectional area of the lumen at the apex of the LSC was measured in a plane perpendicular to the transversal plane of that canal.

For linear measurements of the VA, the Cincinnati protocol was followed (*90*). On the other hand, following the recommendations of Weiss *et al.* (*91*), the volume of the VA was measured, establishing its limits at the intersection of this structure with the vestibule and in the plane perpendicular to the opercular rim.

The measurements of the vestibule (area, height, and width) were taken according to the plane used by Purcell *et al.* (*22*). The volume of the cochlea was measured using the protocol described by Kirk and Gosselin-Ildari (*92*). Regarding cochlear nerve canal width, the protocol described by Yang *et al.* (*93*) was used.

## Supplementary Materials

### This PDF file includes:

Supplementary Text Figs. S1 to S5 Tables S1 to S3 Legends for data S1 to S5 References
