# Supplementary material for: The child who lived: Down syndrome among Neanderthals?
Source: Sci Adv. 2024 Jun 26;10(26):eadn9310. doi: 10.1126/sciadv.adn9310 (PMC11204207; doi:10.1126/sciadv.adn9310)
Supplement: Supplementary file 1 — Supplementary Text Figs. S1 to S5 Tables S1 to S3 Legends for data S1 to S5 References [file sciadv.adn9310_sm.pdf]

Science Advances
AAAS

Supplementary Materials for

The child who lived: Down syndrome among Neanderthals?

Mercedes Conde-Valverde *et al.*

Corresponding author: Mercedes Conde-Valverde, mercedes.conde@uah.es

Sci. Adv. **10**, eadn9310 (2024)
DOI: 10.1126/sciadv.adn9310

The PDF file includes:

Supplementary Text
Figs. S1 to S5
Tables S1 to S3
Legends for data S1 to S5
References

Other Supplementary Material for this manuscript includes the following:

Data S1 to S5

Text

Discriminant analysis

Table S1 shows the basic statistics and the Shapiro-Wilks test of normality of all groups for all variables. We also performed the Box's M test to check the homogeneity of the covariance matrices for those groups (table S2). Once these assumptions have been checked, a discriminant analysis is performed with the four variables. Classification results are shown in the table S3.

83.5% of the original clustered cases are correctly classified. 77.2% of cross-validated clustered cases classified correctly. From those cases, 96.6% of neandertal individuals are corrected classified in both original and Crossing validation. Only Le Moustier 1 is classified as Fossil *Homo sapiens.* One Fossil *Homo sapiens* and one recent *Homo sapiens* are also classified as neandertals, these are Qazfeh 15 (probability of belonging to neandertals = 0.563 original functions and 0.719 in cross validation) and CSJ-50 (P = 0.774 and 0.814 respectively). CN-46700 is assigned to Neanderthals with a probability of 94%.

Recent modern humans are correctly classified in the 80.8% with original functions and 76.9 in cross validation. Fossil modern humans are the less accurate in the classification, being 70.8% and 54.2 in original and cross validation options. In those two groups, the misclassification are between them, and not with Neanderthals, except the ones mentioned above.

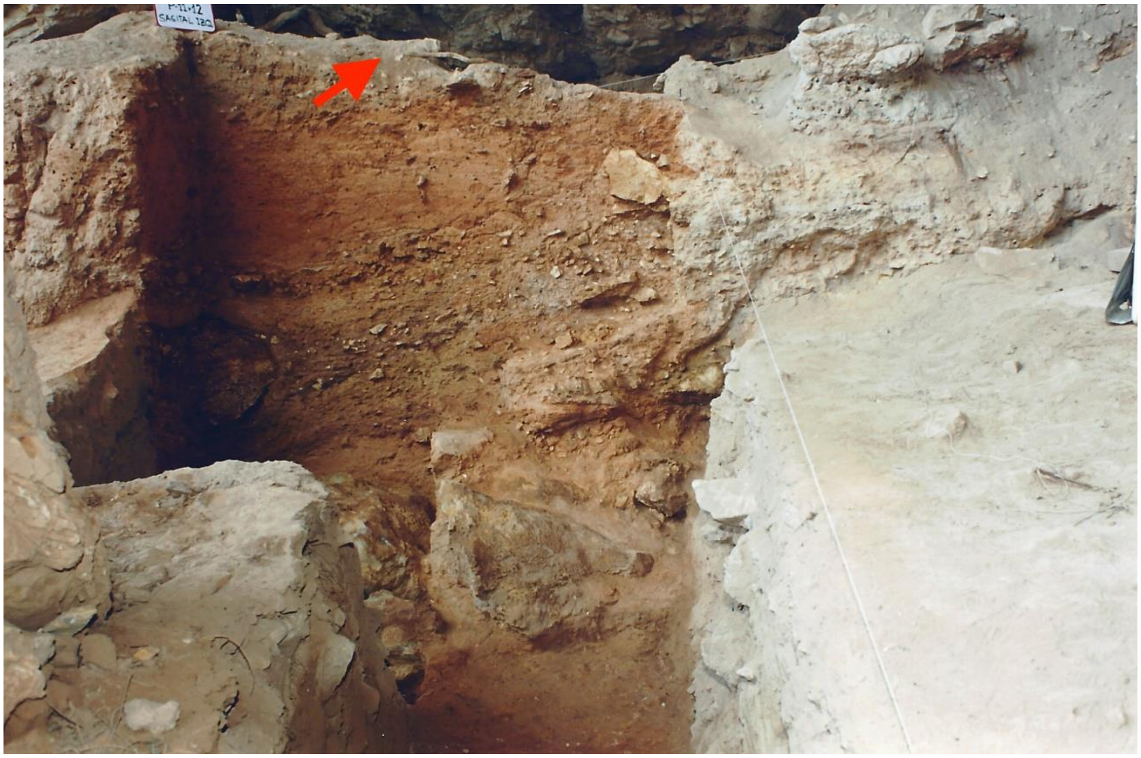

Figure S1. View of the left sagittal section of the south sector (squares P11 and P12) at the end of the 1989 campaign. Red arrow indicates the position of the disturbed surface level, which has not been dated, where CN-46700 has been recovered.

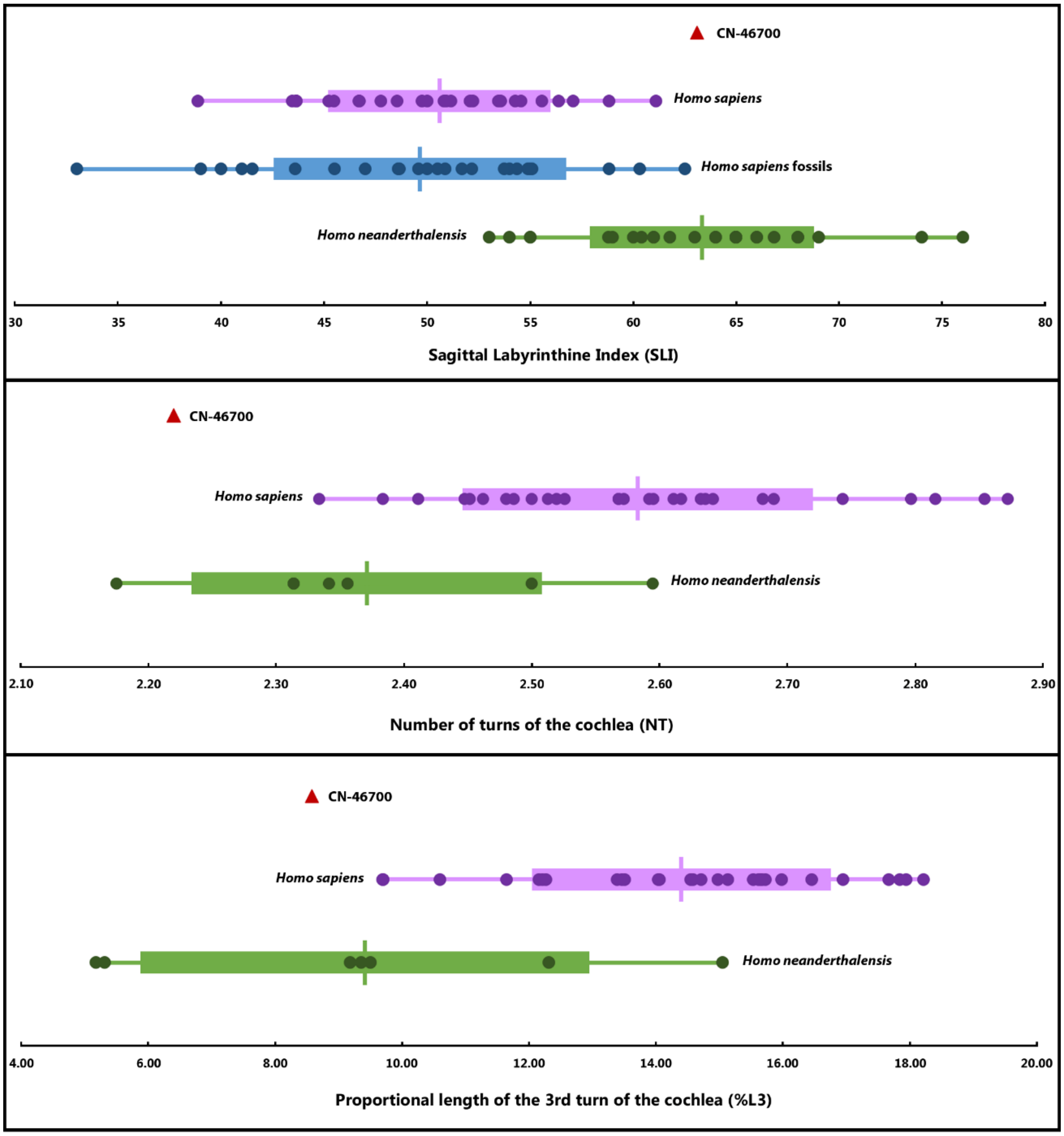

Fig. S2. Range of variation of taxonomically diagnostic inner ear variables in our samples of Neanderthals, *Homo sapiens* and the individual CN-46700. The vertical bar indicates the mean, the thick horizontal bar represents ± 1 standard deviation, and the thin horizontal bar corresponds to the range in each sample.

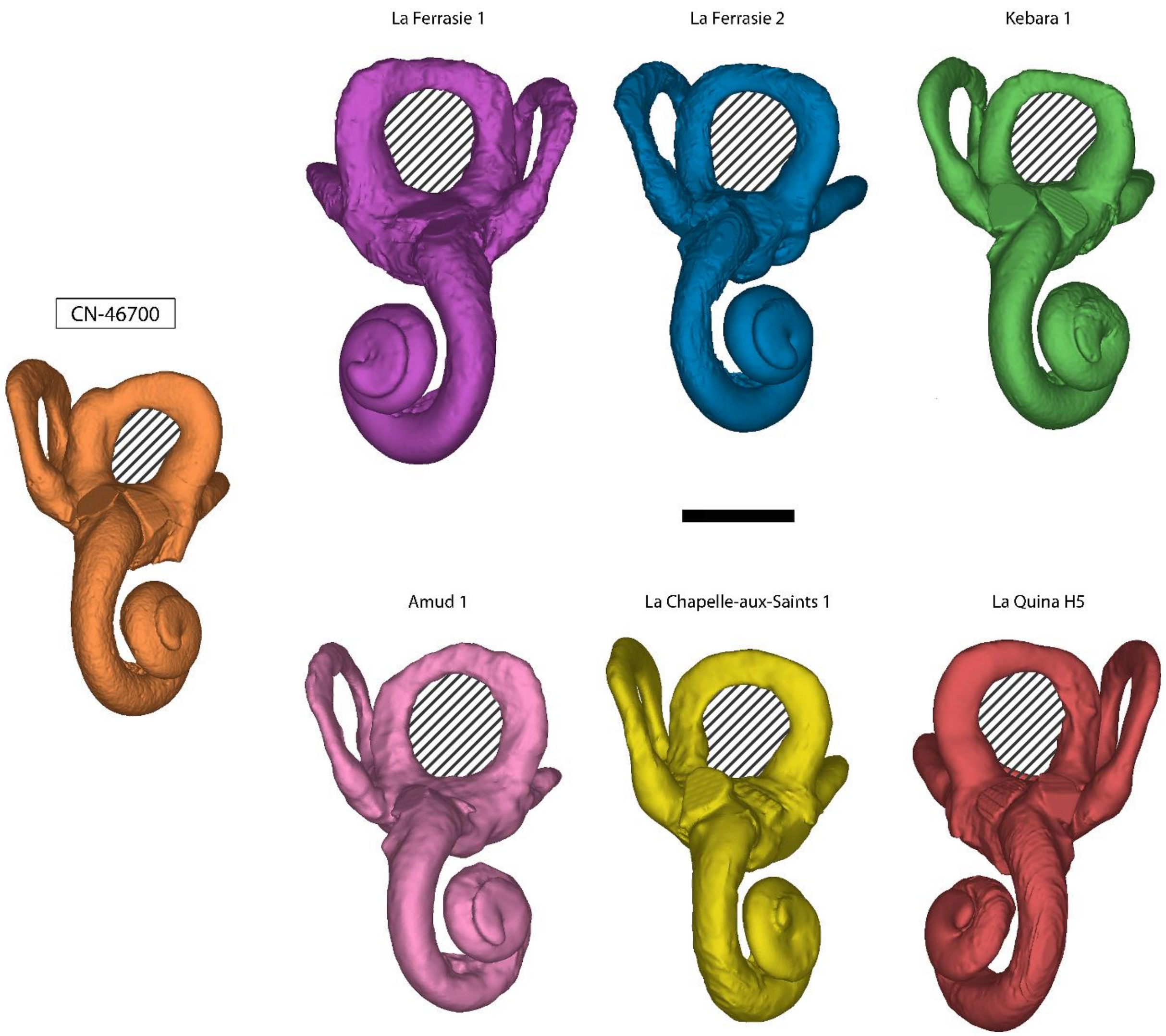

Fig. S3. 3D model of the inner ear of CN-46700 compared to other Neanderthals. The striped area indicates the dimensions of the bony island of CN-46700 compared to other Neandertal specimens in the sample. Scale bar = 5 mm.

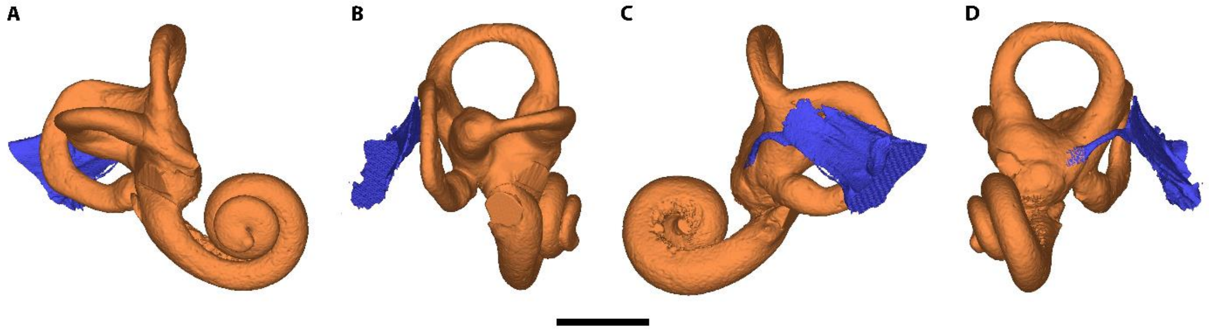

Fig. S4. 3D model of the inner ear (orange) and vestibular aqueduct (blue) of CN-46700. A: anterior view; B: lateral view; C: posterior view; D: medial view. Scale bar = 5 mm.

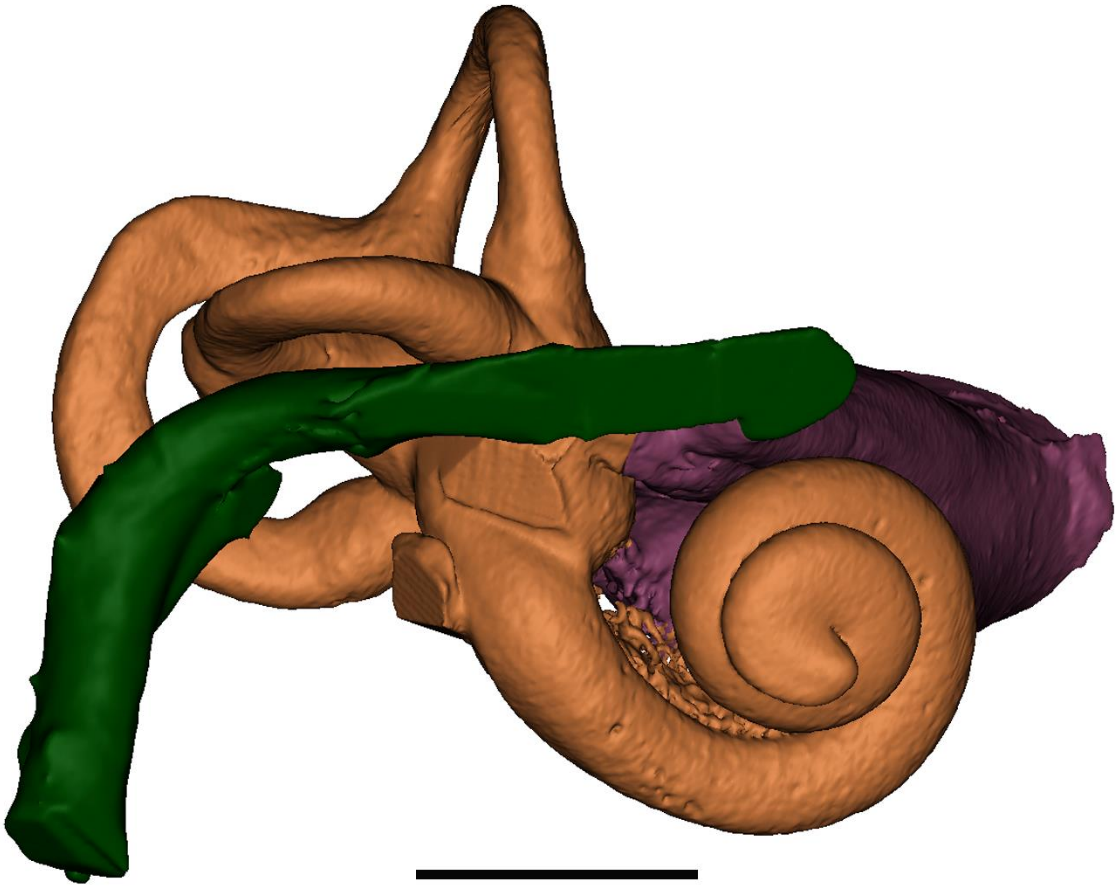

Fig. S5. 3D model of the inner ear and the trajectory of the facial nerve in CN-46700. Inner ear is in orange, internal auditory canal is in magenta, and facial nerve is in green. The facial nerve has a normal trajectory between the lateral semicircular canal and the cochlea. Scale bar = 5mm.

Table S1. Basic statistics and Normality test by groups

		N	Mean	Std. Deviation	Shapiro-Wilk	P-value of Shapiro-Wilk
ASC-R	Fossil *H. sapiens*	25	3.29	0.18	0.946	0.20
	H. neanderthalensis	30	3.02	0.17	0.944	0.12
	Recent *H. sapiens*	26	3.13	0.24	0.975	0.76
PSC-R	Fossil *H. sapiens*	24	3.01	0.20	0.916	0.05
	H. neanderthalensis	29	2.82	0.24	0.966	0.46
	Recent *H. sapiens*	26	3.10	0.26	0.942	0.15
LSC-R	Fossil *H. sapiens*	25	2.46	0.17	0.980	0.89
	H. neanderthalensis	30	2.56	0.15	0.951	0.18
	Recent *H. sapiens*	26	2.32	0.22	0.966	0.53
SLI	Fossil *H. sapiens*	26	49.29	7.20	0.973	0.71
	H. neanderthalensis	29	62.98	5.71	0.959	0.31
	Recent *H. sapiens*	26	50.46	5.34	0.991	1.00

Table S2. Box's M-test for Homogeneity of Covariance Matrices

χ^2	df	p
27.641	20	0.118

Table S3. Discriminant analysis carried out taking into account the number of individuals in each group.

Group		Correct cases			Total
		H. neanderthalensis	Fossil *H. sapiens*	Recent *H. sapiens*	
Original	*H. neanderthalensis*	28	1	0	29
	Fossil *H. sapiens*	1	17	6	24
	Recent *H. sapiens*	1	4	21	26
Cross validation	*H. neanderthalensis*	28	1	0	29
	Fossil *H. sapiens*	1	13	10	24
	Recent *H. sapiens*	1	5	20	26

Data S1. Individual values of number of turns (NT) and proportional length of the third turn (%L3) of the sample use for taxonomical diagnosis.

Separate file.

Data S2. Individual values of the Neanderthal, *Homo sapiens* fossils and recent *Homo sapiens* samples of the taxonomic diagnostic variables of the inner ear.
Separate file.
Radii of curvature of the Anterior Semicircular Canal (ASR-R), Posterior Semicircular Canal (PSC-R), Lateral Semicircular Canal (LSC-R) and Sagittal Labyrinthine Index (SLI).

Data S3. Individual values of the Neanderthal sample of the pathologically diagnostic variables of the inner ear.
Separate file.
LSC: Lateral Semicircular Canal; ASC: Anterior Semicircular Canal; PSC: Posterior Semicircular Canal; VA: Vestibular Aqueduct; V: Vestibule; C: Cochlea; CNC: Cochlear Nerve Canal. *Data from (*104*)

Data S4. Measurement protocol of the pathologically diagnostic variables of the inner ear.
Separate file.
LSC: Lateral Semicircular Canal; ASC: Anterior Semicircular Canal; PSC: Posterior Semicircular Canal; VA: Vestibular Aqueduct; V: Vestibule; C: Cochlea; CNC: Cochlear Nerve Canal

Data S5. 3D model of the inner ear of CN-46700

REFERENCES AND NOTES

1. J. Hublin, The prehistory of compassion. *Proc. Natl. Acad. Sci. U.S.A.* **106**, 6429–6430 (2009).

2. N. Thorpe, “The Palaeolithic Compassion Debate–Alternative projections of modern-day disability into the distant past” in *Care in the Past: Archaeological and Interdisciplinary Perspectives*, L. Powell, W. Southwell-Wright, R. Gowland, Eds. (Oxbow Books, 2016), pp. 91–109.

3. L. Tilley, “Disability and care in the bioarchaeological record. Meeting the challenges of being human” in *The Routledge Handbook of Paleopathology* (Routledge, 2023), pp. 457–481.

4. P. Spikins, “Material Evidence: Caring for adult vulnerabilities” in *Hidden Depths: The Origins of Human Connection* (White Rose Univ. Press, 2022), pp. 71–127.

5. C. P. van Schaik, J. M. Burkart, “Mind the gap: Cooperative breeding and the evolution of our unique features” in *Mind the Gap: Tracing the Origins of Human Universals* (Springer Berlin Heidelberg, 2010), pp. 477–496.

6. L. Tilley, “Setting the scene for a bioarchaeology of care” in *Theory and Practice in the Bioarchaeology of Care* (Springer, 2015), pp. 13–64.

7. P. Spikins, A. Needham, L. Tilley, G. Hitchens, Calculated or caring? Neanderthal healthcare in social context. *World Archaeol.* **50**, 384–403 (2018).

8. R. J. Planer, Cooperative breeding and the evolutionary origins of shared intentionality. *Philos. Theory Practice Biol.* **15**, 2 (2023).

9. K. A. Dettwyler, Can paleopathology provide evidence for “compassion”? *Am. J. Phys. Anthropol.* **84**, 375–384 (1991).

10. D. DeGusta, Comparative skeletal pathology and the case for conspecific care in Middle Pleistocene hominids. *J. Archaeol. Sci.* **29**, 1435–1438 (2002).

11. D. DeGusta, Aubesier 11 is not evidence of Neanderthal conspecific care. *J. Hum. Evol.* **45**, 91–94 (2003).

12. J. M. Burkart, C. van Schaik, M. Griesser, Looking for unity in diversity: Human cooperative childcare in comparative perspective. *Proc. R. Soc. B* **284**, 20171184 (2017).

13. A. Eixea, I. Oltra, M. M. Bergadà, V. Villaverde, The reinterpretation of the Cova Negra archaeological and stratigraphical sequence and its implications in the understanding of the Middle Palaeolithic Iberian Peninsula. *Quat. Int.* **566-567**, 98–112 (2020).

14. M. Richard, C. Falguères, E. Pons-Branchu, L. Foliot, P.M. ESR/U-series chronology of early Neanderthal occupations at Cova Negra (Valencia, Spain). *Quat. Geochronol.* **49**, 283–290 (2019).

15. J. L. Arsuaga, V. Villaverde, R. Quam, I. Martínez, J. M. Carretero, C. Lorenzo, A. Gracia, New Neandertal remains from Cova Negra (Valencia, Spain). *J. Hum. Evol.* **52**, 31–58 (2007).

16. F. Spoor, J. Hublin, M. Braun, F. Zonneveld, The bony labyrinth of Neanderthals. *J. Hum. Evol.* **44**, 141–165 (2003).

17. M. Conde-Valverde, I. Martínez, R. M. Quam, A. Bonmatí, C. Lorenzo, A. D. Velez, C. Martínez-Calvo, J. L. Arsuaga, The cochlea of the Sima de los Huesos hominins (Sierra de Atapuerca, Spain): New insights into cochlear evolution in the genus Homo. *J. Hum. Evol.* **136**, 102641 (2019).

18. H. Coqueugniot, J. J. Hublin, F. Veillon, F. Houët, T. Jacob, Early brain growth in Homo erectus and implications for cognitive ability. *Nature* **431**, 299–302 (2004).

19. M. Maślanka, T. Skadorwa, B. Ciszek, Postnatal development of the subarcuate fossa and subarcuate canaliculus—A computed tomographic study. *Surg. Radiol. Anat.* **40**, 1111–1117 (2018).

20. C. Kenis, M. Ditchfield, E. Paul, P. M. Parizel, S. Stuckey, The petromastoid canal in the young child: Appearance on computed tomography. *Int. J. Pediatr. Otorhinolaryngol.* **77**, 803–807 (2013).

21. A. Rosas, L. Ríos, A. Estalrrich, H. Liversidge, A. García-Tabernero, R. Huguet, H. Cardoso, M. Bastir, C. Lalueza-Fox, M. de la Rasilla, C. Dean, The growth pattern of Neandertals, reconstructed from a juvenile skeleton from El Sidrón (Spain). *Science* **357**, 1282–1287 (2017).

22. D. Purcell, J. Johnson, N. Fischbein, A. K. Lalwani, Establishment of normative cochlear and vestibular measurements to aid in the diagnosis of inner ear malformations. *Otolaryngol. Head Neck Surg.* **128**, 78–87 (2003).

23. M. Y. Lan, J. Y. Shiao, C. Y. Ho, H. C. Hung, Measurements of normal inner ear on computed tomography in children with congenital sensorineural hearing loss. *Eur. Arch. Oto-Rhino-Laryn.* **266**, 1361–1364. (2009).

24. P. J. Govaerts, J. Casselman, K. Daemers, G. De Ceulaer, T. Somers, F. E. Offeciers, Audiological findings in large vestibular aqueduct syndrome. *Int. J. Pediatr. Otorhinolaryngol.* **51**, 157–164 (1999).

25. I. M. Ishida, M. Sugiura, T. Nakashima, S. Naganawa, E. Sato, J. Sugiura, T. Yoshino, Lateral semicircular canal and vertigo in patients with large vestibular aqueduct syndrome. *Otol. Neurotol.* **27**, 788–792 (2006).

26. C. M. Clark, H. H. Patel, S. G. Kanekar, H. Isildak, Enlarged vestibular aqueducts and other inner-ear abnormalities in patients with Down syndrome. *J. Laryngol. Otol.* **131**, 298–302 (2017).

27. M. Suzuki, Y. Ota, T. Tanaka, Y. Ota, Lateral semicircular canal–enlarged vestibular aqueduct fistula associated with paroxysmal positional nystagmus. *Otol. Neurotol.* **37**, e192–e193 (2016).

28. J. L. Sheehy, D. E. Brackmann, Cholesteatoma surgery: Management of the labyrinthine fistula—A report of 97 cases. *Laryngoscope* **89**, 78–87 (1979).

29. L. P. S. Rosito, I. Canali, A. Teixeira, M. N. Silva, F. Selaimen, S. S. Costa, Cholesteatoma labyrinthine fistula: Prevalence and impact. *Braz. J. Otorhinolaryngol.* **85**, 222–227 (2019).

30. L. Sennaroglu, I. Saatci, A new classification for cochleovestibular malformations. *Laryngoscope* **112**, 2230–2241 (2002).

31. S. Blaser, E. J. Propst, D. Martin, A. Feigenbaum, A. L. James, P. Shannon, B. C. Papsin, Inner ear dysplasia is common in children with Down syndrome (trisomy 21). *Laryngoscope* **116**, 2113–2119 (2006).

32. E. J. Propst, S. Blaser, K. A. Gordon, R. V. Harrison, B. C. Papsin, Temporal bone findings on computed tomography imaging in branchio-oto-renal syndrome. *Laryngoscope* **115**, 1855–1862 (2005).

33. B. M. O'Brien, S. P. Meyers, B. T. Crane, Brachio-oto-renal syndrome: CT imaging and intra-operative diagnostic findings. *Otol. Neurotol.* **36**, e110–e111 (2015).

34. S. H. Kwak, M. K. Kim, S. H. Kim, J. Jung, Audiological and vestibular functions in patients with lateral semicircular canal dysplasia and aplasia. *Clin. Exp. Otorhinolaryngol.* **13**, 255–260 (2020).

35. I. Dallan, S. Berrettini, E. Neri, A. P. Casani, Bilateral, isolated, lateral semicircular canal malformation without hearing loss. *J. Laryngol. Otol.* **122**, 858–860 (2008).

36. J. Johnson, A. K. Lalwani, Sensorineural and conductive hearing loss associated with lateral semicircular canal malformation. *Laryngoscope* **110**, 1673–1679 (2000).

37. D. Brotto M. Ariano, M. Sozzi, R. Cenedese, E. Muraro, F. Sorrentino, P. Trevisi, Vestibular anomalies and dysfunctions in children with inner ear malformations: A narrative review. *Front. Pediatr.* **11**, 1027045 (2023).

38. S. Berrettini, F. Forli, F. Bogazzi, E. Neri, L. Salvatori, A. P. Casani, S. S. Franceschini, Large vestibular aqueduct syndrome: Audiological, radiological, clinical, and genetic features. *Am. J. Otolaryngol.* **26**, 363–371 (2005).

39. G. E. Valvassori, J. D. Clemis, The large vestibular aqueduct syndrome. *Laryngoscope* **88**, 723–728 (1978).

40. R. K. Jackler, A. de la Cruz, The large vestibular aqueduct syndrome. *Laryngoscope* **99**, 1238–1243 (1989).

41. V. Maiolo, G. Savastio, G. C. Modugno, L. Barozzi, Relationship between multidetector CT imaging of the vestibular aqueduct and inner ear pathologies. *Neuroradiology J.* **26**, 683–692 (2013).

42. E. Hassanein, M. A. Ghaffar, M. Taha, H. Taha, Clinical significance of vestibular aqueduct's width in pediatric patients with sensorineural hearing loss. *Mediterr. J. Otol.* **4**, 109–117 (2008).

43. J. F. Grimmer, G. Hedlund, Vestibular symptoms in children with enlarged vestibular aqueduct anomaly. *Int. J. Pediatr. Otorhinolaryngol.* **71**, 275–282 (2007).

44. J. Song, S. Hong, S. Lee, S. Park, S. Kang, Y. An, J. Jang, J. Kim, J. Koo, Vestibular manifestations in subjects with enlarged vestibular aqueduct. *Otol. Neurotol.* **39**, e461–e467 (2018).

45. F. C. Sarioglu, Y. Pekcevik, H. Guleryuz, A. C. Cetin, E. A. Guneri, The relationship between the third window abnormalities and inner ear malformations in children with hearing Loss. *J. Int. Adv. Otol.* **17**, 387–392 (2021).

46. P. A. Wackym, Y. Agrawal, T. Ikezono, C. D. Balaban, Editorial: Third window syndrome. *Front. Neurol.* **12**, 704095 (2021).

47. K. D. Blake, C. Prasad, CHARGE syndrome. *Orphanet J. Rare Dis.* **1**, 34 (2006).

48. P. D. Phelps, R. A. Coffey, R. C. Trembath, L. M. Luxon, A. B. Grossman, K. E. Britton, P. Kendall-Taylor, J. M. Graham, B. C. Cadge, S. G. D. Stephens, M. E. Pembrey, W. Reardon, Radiological malformations of the ear in Pendred syndrome. *Clin. Radiol.* **53**, 268–273 (1998).

49. R. J. Smith, Y. Iwasa, A. M. Schaefer, "Pendred syndrome/nonsyndromic enlarged vestibular aqueduct" in *GeneReviews®*, M. P. Adam, H. H. Ardinger, R. A. Pagon, S. E. Wallace, L. J. H. Bean, K. Stephens, A. Amemiya, Eds. (University of Washington, 2020), pp.1993–2019.

50. G. Kontorinis, F. Goetz, H. Lanfermann, S. Luytenski, A. M. Giesemann, Inner ear anatomy in Waardenburg syndrome: Radiological assessment and comparison with normative data. *Int. J. Pediatr. Otorhinolaryngol.* **78**, 1320–1326 (2014).

51. T. Okuno, H. Takahashi, Y. Shibahara, Y. Hashida, I. Sando, Temporal bone histopathologic findings in Alagille's syndrome. *Arch. Otolaryngol. Head Neck Surg.* **116**, 217–220 (1990).

52. A. K. Morimoto, R. H. Wiggins III, P. A. Hudgins, G. L. Hedlund, B. Hamilton, S. K. Mukherji, S. A. Telian, H. R. Harnsberger, Absent semicircular canals in CHARGE syndrome: Radiologic spectrum of findings. *Am. J. Neuroradiol.* **27**, 1663–1671 (2006).

53. A. C. Vesseur, B. M. Verbist, H. E. Westerlaan, F. J. J. Kloostra, R. J. C. Admiraal, C. M. A. van Ravenswaaij-Arts, R. H. Free, E. A. M. Mylanus, CT findings of the temporal bone in CHARGE syndrome: Aspects of importance in cochlear implant surgery. *Eur. Arch. Otorhinolaryngol.* **273**, 4225–4240 (2016).

54. M. Elmaleh-Bergès, C. Baumann, N. Noël-Pétroff, A. Sekkal, V. Couloigner, K. Devriendt, M. Wilson, S. Marlin, G. Sebag, V. Pingault, Spectrum of temporal bone abnormalities in patients with Waardenburg syndrome and SOX10 mutations. *Am. J. Neuroradiol.* **34**, 1257–1263 (2013).

55. K. S. Traylor, S. Dodson, S. F. Kralik, C. Ho, R. Radhakrishnan, Inner ear anomalies in congenital hearing loss: Imaging, genetics, and associated syndromes. *Neurographics* **9**, 358–372 (2019).

56. P. Feraco, S. Piccinini, C. Gagliardo, Imaging of inner ear malformations: A primer for radiologists. *Radiol. Med.* **126**, 1282–1295 (2021).

57. M. Igarashi, M. Takahashi, B. R. Alford, P. E. Johnson, Inner ear morphology in Down's syndrome. *Acta Otolaryngol.* **83**, 175–181 (1977).

58. C. J. Chin, M. M. Khami, M. Husein, A general review of the otolaryngologic manifestations of Down syndrome. *Int. J. Pediatr. Otorhinolaryngol.* **78**, 899–904 (2014).

59. T. Inagaki, N. Morita, S. Cureoglu, P. A. Schachern, S. Nomiya, R. Nomiya, M. M. Paparella, Peripheral vestibular system in Down syndrome: Quantitative assessment of vestibular histopathology. *Otolaryngol. Head Neck Surg.* **144**, 280–283 (2011).

60. M. J. Bull, Down Syndrome. *N. Engl. J. Med.* **382**, 2344–2352 (2020).

61. J. M. Starbuck, On the antiquity of trisomy 21: Moving towards a quantitative diagnosis of down syndrome in historic material culture. *J. Contemp. Anthropol.* **2**, 19–44 (2011).

62. T. Matsumoto, N. Itoh, S. Inoue, M. Nakamura, An observation of a severely disabled infant chimpanzee in the wild and her interactions with her mother. *Primates* **57**, 3–7 (2016).

63. R. S. Chapman, L. J. Hesketh, Behavioral phenotype of individuals with Down syndrome. *Ment. Retard. Dev. Disabil. Res. Rev.* **6**, 84–95 (2000).

64. W. Silverman, Down syndrome: Cognitive phenotype. *Ment. Retard. Dev. Disabil. Res. Rev.* **13**, 228–236 (2007).

65. J. Grieco, M. Pulsifer, K. Seligsohn, B. Skotko, A. Schwartz, Down syndrome: Cognitive and behavioral functioning across the lifespan. *Am. J. Med. Genet. C Semin. Med. Genet.* **169C**, 135–149 (2015).

66. A. C. Cardoso, A. C. de Campos, M. M. Dos Santos, D. C. C. Santos, N. A. C. F. Rocha, Motor performance of children with Down syndrome and typical development at 2 to 4 and 26 months. *Pediatr. Phys. Ther.* **27**, 135–141 (2015).

67. G. Laws, A. Hall, Early hearing loss and language abilities in children with Down syndrome. *Int. J. Lang. Comm. Dis.* **49**, 333–342 (2014).

68. S. N. Van Cleve, W. I. Cohen, Part I: clinical practice guidelines for children with Down syndrome from birth to 12 years. *J. Pediatr. Health Care* **20**, 47–54 (2006).

69. R. Ranweiler, Assessment and care of the newborn with Down syndrome. *Adv. Neonatal Care* **9**, 17–24 (2009).

70. K. Mizuno, A. Ueda, Development of sucking behavior in infants with Down's syndrome. *Acta Paediatr.* **90**, 1384–1388 (2001).

71. M. J. Aguilar-Cordero, R. Rodríguez-Blanque, A. Sánchez-López, X. A. León-Ríos, M. Expósito-Ruiz, N. Mur-Villar, Assessment of the technique of breastfeeding in babies with down syndrome. *Aquichan.* **19**, 1–12 (2019).

72. V. S. Coentro, D. T. Geddes, S. L. Perrella, Altered sucking dynamics in a breastfed infant with Down syndrome: A case report. *Int. Breastfeed. J.* **15**, 71 (2020).

73. I. R. Agulló, B. M. González, Factores que influyen en el desarrollo motor de los niños con síndrome de Down. *Rev. Med. Int. Sindr. Down.* **10**, 18–24 (2006).

74. R. J. Palisano, S. D. Walter, D. J. Russell, P. L. Rosenbaum, M. Gémus, B. E. Galuppi, L. Cunningham, Gross motor function of children with Down syndrome: Creation of motor growth curves. *Arch. Phys. Med. Rehabil.* **82**, 494–500 (2001).

75. M. Alesi, G. Battaglia, Motor development and Down syndrome. *Int. Rev. Res. Dev. Disabil.* **56**, 169–211 (2019).

76. P. D. Jain, A. Nayak, S. D. Karnad, K. N. Doctor, Gross motor dysfunction and balance impairments in children and adolescents with Down syndrome: A systematic review. *Clin. Exp. Pediatr.* **65**, 142–149 (2022).

77. M. S. Van der Linden, S. Vucic, D. Van Marrewijk, E. M. Ongkosuwito, Dental development in Down syndrome and healthy children: A comparative study using the Demirjian method. *J. Orthod. Craniofac. Res.* **20**, 65–70 (2017).

78. A.B. Rohrlach, M. Rivollat, P. de-Miguel-Ibáñez, U. Moilanen, A. M. Liira, J. C. Teixeira, X. Roca-Rada, J. Armendáriz-Martija, K. Boyadzhiev, Y. Boyadzhiev, B. Llamas, A. Tiliakou, A. Mötsch, J. Tuke, E. A. Prevedorou, N. Polychronakou-Sgouritsa, J. Buikstra, P. Onkamo, P. W. Stockhammer, H. O. Heyne, J. R. Lemke, R. Risch, S. Schiffels, J. Krause, W. Haak, K. Prüfer, Cases of trisomy 21 and trisomy 18 among historic and prehistoric individuals discovered from ancient DNA. *Nat. Commun.* **15**, 1294 (2024).

79. A. H. Bittles, E. Glasson, Clinical, social, and ethical implications of changing life expectancy in Down syndrome. *Dev. Med. Child Neurol.* **46**, 282–286 (2004).

80. F. Spoor, C. Stringer, F. Zonneveld, Rare temporal bone pathology of the Singa calvaria from Sudan. *Am. J. Phys. Anthropol.* **107**, 41–50 (1998)

81. D. Coutinho-Nogueira, H. Coqueugniot, O. Dutour, A. Ben-Ncer, J. Hublin, Inner ear modifications in Dar-es-Soltane II H5 (Morocco): A case of labyrinthitis ossificans. *Int. J. Paleopathol.* **38**, 41–44 (2022).

82. H. Coqueugniot, O. Dutour, B. Arensburg, H. Duday, B. Vandermeersch, A. Tillier, Earliest cranio-encephalic trauma from the levantine middle palaeolithic: 3D reappraisal of the Qafzeh 11 skull, consequences of pediatric brain damage on individual life condition and social care. *PLOS ONE* **9**, e102822 (2014).

83. A. Gracia, J. L. Arsuaga, I. Martínez, C. Lorenzo, J. M. Carretero, J. M. Bermúdez de Castro, E. Carbonell, Craniosynostosis in the middle pleistocene human cranium 14 from the Sima de los Huesos, Atapuerca, Spain. *Proc. Natl. Acad. Sci. USA.* **106**, 6573–6578 (2009).

84. R. Grün, C. B. Stringer, Electron spin resonance dating and the evolution of modern humans. *Archaeometry*. **33**, 153–199 (1991).

85. M. Frouin, C. Lahaye, H. Valladas, T. Higham, A. Debénath, A. Delagnes, N. Mercier, Dating the middle paleolithic deposits of La Quina Amont (Charente, France) using luminescence methods. *J. Hum. Evol.*, **109**, 30–45 (2017).

86. G. Guérin, M. Frouin, S. Talamo, V. Aldeias, L. Bruxelles, L. Chiotti, H. L. Dibble, P. Goldberg, J. Hublin, M. Jain, C. Lahaye, S. Madelaine, B. Maureille, S. J. P. McPherron, N. Mercier, A. S. Murray, D. Sandgathe, T. E. Steele, K. J. Thomsen, A. Turq, A multi-method luminescence dating of the Palaeolithic sequence of La Ferrassie based on new excavations adjacent to the La Ferrassie 1 and 2 skeletons. *J. Archaeol. Sci.* **58**, 147–166 (2015).

87. W. J. Rink, H. P. Schwarcz, H. K. Lee, J. Rees-Jones, R. Rabinovich, E. Hovers, Electron spin resonance (ESR) and thermal ionization mass spectrometric (TIMS)230Th/234U dating of teeth in Middle Paleolithic layers at Amud Cave, Israel. *Geoarchaeology* **16**, 701–717 (2001).

88. H. P. Schwarcz, W. M. Buhay, R. Grün, H. Valladas, E. Tchernov, O. Bar-Yosef, B. Vandermeersch, ESR dating of the Neanderthal site, Kebara Cave, Israel. *J. Archaeol. Sci.* **16**, 653–659 (1989).

89. J. Skrzat, B. Leszczyński, M. Kozerska, A. Wróbel, Topography and morphometry of the subarcuate canal. *Folia Morphol.* **72**, 357–361 (2013).

90. M. Boston, M. Halsted, J. Meinzen-Derr, J. Bean, S. Vijayasekaran, E. Arjmand, D. Choo, C. Benton, J. Greinwald, The large vestibular aqueduct: A new definition based on audiologic and computed tomography correlation. *Otolaryngol. Head Neck Surg.* **136**, 972–977 (2007).

91. N. M. Weiss, T. M. Breitsprecher, A. Pscheidl, D. Bächinger, S. Volkenstein, S. Dazert, R. Mlynski, S. Langner, P. Roland, A. Dhanasingh, Volumetry improves the assessment of the vestibular aqueduct size in inner ear malformation. *Eur. Arch. Otorhinolaryngol.* **280**, 2155–2163 (2023).

92. E. C. Kirk, A. D. Gosselin-Ildari, Cochlear labyrinth volume and hearing abilities in primates. *Anat. Rec.* **292**, 765–776 (2009).

93. F. Yan, J. Li, J. Xian, Z. Wang, L. Mo, The cochlear nerve canal and internal auditory canal in children with normal cochlea but cochlear nerve deficiency. *Acta Radiol.* **54**, 292–298 (2013).

94. R. Quam, C. Lorenzo, I. Martínez, A. Gracia-Téllez, J. L. Arsuaga, The bony labyrinth of the middle Pleistocene Sima de los Huesos hominins (Sierra de Atapuerca, Spain). *J. Hum. Evol.* **90**, 1–15 (2016).

95. A. Uhl, H. Reyes-Centeno, D. Grigorescu, E. F. Kranioti, K. Harvati, Inner ear morphology of the cioclovina early modern European Calvaria from Romania. *Am. J. Phys. Anthropol.* **160**, 62–70 (2016).

96. J.J. Hublin, F. Spoor, M. Braun, F. Zonneveld, S. Condemi, A late Neanderthal associated with Upper Palaeolithic artefacts. *Nature* **381**, 224–226 (1996).

97. A. Gómez-Olivencia, I. Crevecoeur, A. Balzeau, La Ferrassie 8 Neandertal child reloaded: New remains and re-assessment of the original collection. *J. Hum. Evol.* **82**, 107–126 (2015).

98. M. Glantz, B. Viola, P. Wrinn, T. Chikisheva, A. Derevianko, A. Krivoshapkin, U. Islamov, R. Suleimanov, T. Ritzman, New hominin remains from Uzbekistan. *J. Hum. Evol.* **55**, 223–237 (2015).

99. C. A. Hill, J. Radovčić, D. W. Frayer, Brief communication: Investigation of the semicircular canal variation in the Krapina Neandertals. *Am. J. Phys. Anthropol.* **154**, 302–306 (2014).

100. D. Coutinho-Nogueira, H. Coqueugniot, F. Santos, A. M. Tillier, The bony labyrinth of Qafzeh 25 *Homo sapiens* from Israel. *Archaeol. Anthropol. Sci.* **13**, 151 (2021).

101. X. J. Wu, I. Crevecoeur, W. Liu, S. Xing, E. Trinkaus, Temporal labyrinths of eastern Eurasian Pleistocene humans. *Proc. Natl. Acad. Sci. U.S.A.* **111**, 10509–10513 (2014).

102. M. Ponce De León, C. Zollikofer, "The labyrinthine morphology" in *The Prehistory and Paleontology of the Peştera Muierii, Romania*, A. Doboş¸ A. Soficaru, E. Trinkaus, Eds. (Etud Rech Archeol Univ Liège, 2010), pp. 96–97.

103. I. Crevecoeur, A. Thibeault, L. Bouchneb, M. Matu, B. Maureille, I. Ribot, "The Hofmeyr bony labyrinth: Morphological description and affinity" in *Hofmeyr: A Late Pleistocene Human Skull from South Africa*, F. E. Grine, Ed. (Springer Nature, 2022), pp. 165–178.

104. M. Beals, "The cochlear labyrinth of Krapina Neandertals," thesis, University of Kansas, Kansas (2012).
